# Highly sensitive and selective detection of KLK4 in human serum with a low-cost electrochemical biosensor

**DOI:** 10.55730/1300-0527.3737

**Published:** 2025-04-29

**Authors:** Elif Burcu AYDIN

**Affiliations:** Scientific and Technological Research Center, Tekirdağ Namık Kemal University, Tekirdağ, Turkiye

**Keywords:** Kallikrein 4, prostate cancer, impedimetric biosensor, linear polymer

## Abstract

An ultrasensitive impedimetric immunosensor was fabricated using a poly(glycidyl methacrylate) (PGMA) polymer-covered indium tin oxide (ITO) platform for the quantification of kallikrein 4 (KLK4), an important prostate cancer biomarker. PGMA had suitable biocompatibility and nontoxicity for loading of antiKLK4 antibodies on the ITO substrate surface. Anti-KLK4 biomolecules were directly attached to the PGMA-covered electrode surface via epoxy groups of the PGMA polymer. The preparation method for the PGMA matrix-modified electrode was simple and inexpensive. The proposed biosensor immobilization layers coated on the ITO electrode were characterized with electrochemical techniques. The experimental parameters that affect biosensor response were optimized, and the suggested sensor showed a linear response from 0.04 pg/mL to 8 pg/mL with a low detection limit (LOD) of 12.21 fg/mL. Moreover, it had acceptable stability, reproducibility, and repeatability. Additionally, the disposable biosensor offered excellent reliability and accuracy in KLK4 analysis, suggesting that it could be used as an alternative technique in clinical diagnosis.

## 1. Introduction

The third biggest reason for cancer-related deaths among men worldwide is prostate cancer (PCa) [1,2]. PCa begins in the outermost part of the prostate. Several epigenetic alterations, uncontrolled proliferation, differentiation, and invasion can occur. Over time, PCa can metastasize to different locations in the body, causing morbidity and mortality. The most important intervention to lower PCa mortality risk is early and precise diagnosis. A popular early detection method for PCa is the quantitative assessment of ultralow levels of PCa-specific biomarkers. When it comes to PCa monitoring, diagnosis, prognosis, and treatment, these biomarkers are crucial [3,4]. A prostate-specific antigen (PSA) biomarker level higher than 4 ng/mL is abnormal but can also be associated with benign prostatic hyperplasia, prostatitis, and cystitis. Thus, PSA is deprived of specificity to accurately analyze the presence of PCa. New biomarkers are needed to improve PCa detection efficiency [5,6].

Kallikrein 4 (KLK4) is a member of the kallikrein family and is found in human tissues [7]. It is localized in the cell nucleus, particularly in prostate tissue, and controlled by androgens [8,9]. Compared to a normal prostate, a cancerous prostate has a substantial overexpression of the KLK4 protein [7]. KLK4 is expressed in 100% of PCa cases [7]. In PCa patients, the KLK4 antigen levels have been measured as ≤40 pg/mL [10]. KLK4 is a potential biomarker for improving PCa detection and screening [11,12]. Successful measurement of the KLK4 biomarker in body fluids is important in the diagnosis and treatment monitoring of PCa. Enzyme-linked immunosorbent assay (ELISA) tests are frequently used for the measurement of KLK4 in body fluids. However, this traditional approach is expensive and time-consuming. Additionally, detection needs to be carried out in a laboratory that is properly equipped [13]. The biosensor proposed in the current study facilitates simple and inexpensive measurement of KLK4 concentration. Very few biosensor systems have been developed for KLK4 measurement, so the current study contributes to improving survival rates among PCa patients.

Electrochemical biosensors use biomolecules to identify target analytes. When targets are recognized, electrochemical responses are formed. This type of biosensor has unmatched superiority in the detection of a wide range of target molecules due to high sensitivity, suitable selectivity, affordability, and speed [13,14]. Among different electrochemical techniques, electrochemical impedance spectroscopy (EIS) is a sensitive, label-free detection technique for different types of biosensing events occurring on the electrode surface [15,16]. With the use of the EIS technique, electrochemical processes, diffusion kinetics, and mass transport parameters can be investigated and monitored. The electrochemical impedance is a sum of real (Z_re_) and the imaginary (Z_im_) parts that measure the resistance and capacitance of an electrochemical cell, respectively. Bode and Nyquist plots are two types of impedance plots used to follow the interactions present on the electrode surface [17,18].

Polymers are effective as biomolecule immobilization platforms. They offer several advantages as matrices for biosensing events because of their flexible, inexpensive, and biocompatible properties. Additionally, they are suitable immobilization platforms for covalent coupling of biomolecules to the electrode surface [19]. Because of these advantages, polymers are extensively used in biosensor construction, biomedical applications, delivery, and cell studies [20,21]. The poly(glycidyl methacrylate) PGMA polymer bears numerous epoxy groups and provides a uniform coating on flat surfaces [22]. This makes them a suitable matrix is a prerequisite for sensitive detection of target analytes. Furthermore, PGMA has good operational stability, a large surface area, and is biocompatible [23]. In order to form a polymer matrix material, several procedures including electrochemical polymerization, drop casting, nanocomposite creation, and polymer covering, have been employed for the preparation of electrochemical biosensing systems [24,25]. A straightforward method for preparing working electrode surfaces is spin coating. In spin coating, uniformly thin layers are formed on the surface of the working electrode as it rotates [26,27].

In this study, a new impedimetric biosensor was constructed with the use of a linear PGMA polymer for ultrasensitive determination of KLK4 PCa marker in real serum. For the fabrication of the biosensor, the spin coating strategy was chosen. This technique produced a reproducible, uniform film on the indium tin oxide (ITO) surface. The PGMA polymer used as a matrix in the biosensor production process had suitable biocompatibility and toxicity for the loading of biomolecules. The covalent bonding of biomolecules directly to epoxy groups of PGMA without the need for any other reactant and the use of spin coating as an electrode fabrication process made the preparation of the biosensor simple. Linear PGMA polymer with epoxy ends provided attachment points for anti-KLK4 antibody immobilization. The high specificity of anti-KLK4 antibodies made the biosensor highly sensitive and selective. The strong binding of specific antibodies to KLK4 antigens showed the importance of the interface matrix and its role in the performance of the biosensor. The biosensor was optimized and when used on serum samples, acceptable recovery ranges were obtained.

## 2. Materials and methods

### 2.1. Chemicals and reagents

ITO plates (1 × 0.5 cm), anti-KLK4 human antibodies, KLK4 human antigens, bovine serum albumin (BSA), prostate-specific membrane human antigen (PSMA), interleukin 6 human antigen (IL6), CC chemokine receptor 4 human antigen (CCR4), tumor necrosis factor alpha antigen (TNFα), potassium ferricyanide (III) (K_3_Fe(CN)_6_), potassium ferrocyanide(II) trihydrate (K_4_Fe(CN)_6_), potassium phosphate monobasic (K_2_HPO_4_), potassium phosphate dibasic (KH_2_PO_4_), potassium chloride (KCl), and PGMA were supplied by Sigma-Aldrich. The 50 mM phosphate buffer (pH 7.4) was used to make protein solutions. The redox couple solution [Fe(CN)_6_]^3−/4−^ was used for electrochemical monitoring of this system.

### 2.2. Instrument conditions

All electrochemical analyses were carried out on a Gamry Interface 1000 electrochemical workstation (Warminster, Pennsylvania, USA) with three electrodes: a platinum auxiliary wire, an Ag/AgCl reference electrode, and a disposable ITO working electrode. The electrochemical analyses were performed in a background solution. The EIS analysis range was from 0.05 Hz to 50,000 Hz. The AC and DC voltages were 5 mV and 0 V, respectively. Cyclic voltammetry (CV) analysis range was from −0.5 V to 1 V at a potential scan rate of 100 mV/s.

A spin coater (PVTC-50, MTI Corporation, Richmond, California, USA) was used for biosensor preparation. The morphologies of modified ITO electrodes were analyzed by scanning electron microscopy (SEM, Quanta FEG 250, FEI, Hillsboro, Oregon, USA) and atomic force microscopy (AFM, AFM PLUS+, NanoMagnetic, Ankara, Türkiye). In addition, Fourier-transform infrared spectroscopy (FTIR) analysis (Vertex 70, Bruker, Karlsruhe, Germany) was used to confirm the successful fabrication of bioelectrodes.

### 2.3. Manufacture of disposable electrodes

Scheme illustrates the construction procedure of the sensing system. First, the working electrode was cleaned by washing ultrasonically with acetone, soap solution, and ultrapure water separately for 8 min each and then drying under nitrogen. Before each experiment, the clean electrode was coated with PGMA polymer by using a spin coating process (1 mg/mL polymer solution, 1000 rpm for 1 min spinning time). Before incubation in protein solution, the prepared electrode was washed with ultrapure water. The polymer-covered ITO was then immersed for 45 min in an anti-KLK4 solution to create an ITO/PGMA/anti-KLK4 bioelectrode. After this process, the electrodes were rinsed with ultrapure water. Anti-KLK4-coated electrodes were immersed in BSA solution to remove free epoxy groups. After blockage of the free ends, the bioelectrode was incubated in KLK4 solution for immobilization of KLK4. The constructed electrodes were stored at 4 °C before use.

### 2.4. Optimization and testing of the biosensor

To achieve the optimum sensing signal, different parameters such as the concentration of PGMA and anti-KLK4 antibodies, incubation time of anti-KLK4 and KLK4 antigen were tested. For optimizing PGMA and antibody concentration, different amounts of materials were used for biosensor fabrication, and the maximum response was recorded to determine the optimum concentrations. For optimizing incubation times, electrodes were incubated at different times until the maximum impedimetric response was measured.

### 2.5. Detection of KLK4 antigen in spiked real samples using fabricated immunosensor

Real serum samples were purchased from Sigma-Aldrich. To monitor the matrix effects, the fabricated immunosensors were tested on real human serum samples spiked with 1 and 4 pg/mL of KLK4. Before electrochemical analysis of these spiked samples, they were diluted 10-fold with phosphate buffer. After the analysis of these samples, the recovery rates were calculated.

## 3. Results and discussion

The biosensor fabrication and immobilization process is summarized in Scheme. The procedure has four steps. In Step 1, the PGMA solution (1 mg/mL prepared in acetone) was spread equally onto the ITO platform with a spin coater (1000 rpm for 1 min), and the electrode was dried at room temperature. Step 2 was the immobilization of recognition anti-KLK4 antibodies. Step 3 was the blockage of unbound epoxy groups present on the electrode surface. Finally, Step 4 was the identification of the target KLK4 antigen.

### 3.1. Chemical analysis of the fabricated bioelectrodes

**Figure 1:**
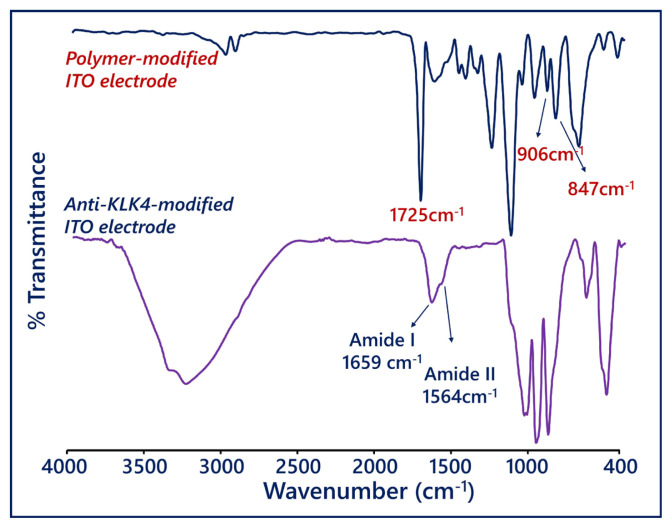
FTIR spectra of ITO/PGMA electrode and ITO/PGMA/antiKLK4 bioelectrode.

The FTIR spectra of the PGMA-covered electrode and the antiKLK4-attached electrode provided information on the successful modification of the electrodes (Figure 1). The peaks of epoxy side ends present on the ITO surface were observed at 907 cm^−1^ and 848 cm^−1^ [28,29]. The peak of carbonyl groups in the repeating unit was observed at 1725 cm^−1^ [30]. The IR spectrum of the PGMA-modified electrode was in accordance with the literature [31]. After binding of anti-KLK4 antibodies onto the PGMA-covered ITO working electrode, two peaks were seen at 1659 cm^−1^ (amide I) and 1564 cm^−1^ (amide II). These bands were the protein-characterized bands [32–34], illustrating that anti-KLK4 was effectively immobilized on PGMA film.

### 3.2. Electrochemical analysis of the biosensor construction process

**Figure 2:**
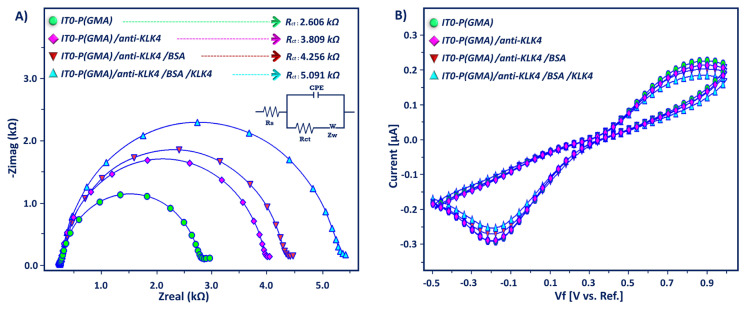
Impedance spectra (A) and CVs (B) of modified ITO electrode in redox couple solution.

EIS analysis was used for determining the relative change in surface charge resistance. Figure 2A shows the impedance spectra displayed as Nyquist curves attained by various modified electrodes in a solution of 5 mM ferri-ferro solution. The electron transfer resistance (R_ct_) at the working electrode surface was calculated by using the Randles equivalent circuit with Gamry software and used to measure the electrical properties of the coating. The Randles equivalent circuit has four elements: Warburg element (W), solution resistance (R_s_), constant phase element (CPE), and R_ct_ [35,36]. The Randles equivalent circuit was used to fit the data recorded over the measured frequency range (Figure 2A inset) sufficiently. The PGMA polymer-covered electrode had a smaller semicircle diameter (2606 Ω) than other fabrication steps. When anti-KLK4 proteins were coated onto the modified electrode, R_ct_ (3809 Ω) increased due to the nonconductive property of proteins; they blocked the electron transfer. After blockage with BSA, the R_ct_ (4256 Ω) increased for the same reason as the anti-KLK4 antibody. BSA proteins created an obstacle to the charge transfer of the redox probe at the electrode interface.

KLK4 antigen immobilization hindered electron transfer, and a large Nyquist plot was obtained (R_ct_, 5091 Ω). Apart from EIS analysis, CV analysis was performed to screen the fabrication procedure. The peak currents at the polymer-coated electrode were high. After coupling of anti-KLK4 antibodies, the CV curves decreased due to slow electron transfer kinetics. The blockage of free epoxy groups with BSA caused decreases in peak currents. This indicates that BSA molecules hinder electron transfer. After KLK4 antigen immobilization, a small peak current was obtained due to the insulating layer of KLK4.

### 3.3. Morphological analysis of the biosensor fabrication steps

**Figure 3:**
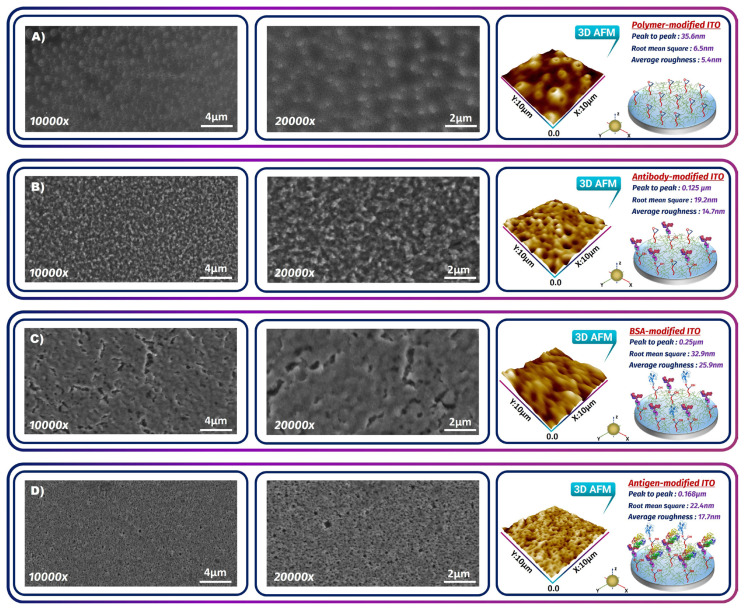
SEM (left) and AFM (right) pictures of functionalized electrode surfaces for (A) polymer-modified ITO, (B) antibody-modified ITO, (C) BSA-modified ITO, and (D) antigen-modified ITO.

SEM and AFM photography revealed the morphological variations present on the working electrode surface during the sensor construction stages. The surface of the electrode coated with PGMA polymer was uniform (Figure 3A). The surface was smooth with a low surface average roughness (5.4 nm).

When anti-KLK4 protein was immobilized on the electrode surface, the SEM image of the surface changed. Figure 3B illustrates globular anti-KLK4 antibodies on the surface. The average roughness increased on the ITO surface to 14.7 nm. The electrode surface was altered by BSA blocking, and the image looked like a classical BSA image (Figure 3C). With the immobilization of BSA molecules, the average roughness increased to 25.9 nm. After KLK4 antigen immobilization, KLK4 biomolecules were found on the modified surface due to specific interaction (Figure 3D). Following this contact, a relatively low average roughness of 17.7 nm was measured, resulting in a smooth surface.

### 3.4. Optimization of experimental variables

**Figure 4:**
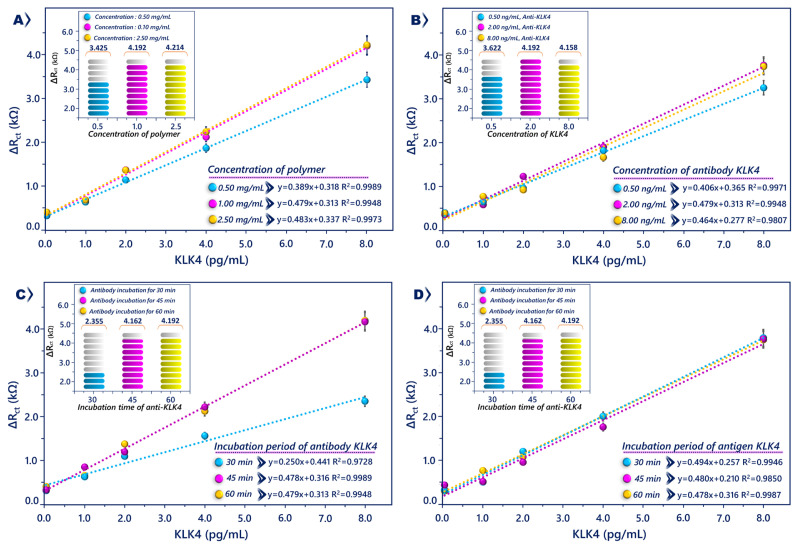
Optimization results of PGMA (A) and anti-KLK4 antibody (B) concentration, and anti-KLK4 (C) and KLK4 (D) incubation time.

Before analytical characterization, the concentration of polymer and antibody, and the incubation time with antibody and antigen, were optimized. Biosensors were prepared using different concentrations (0.5, 1, and 2.5 mg/mL) of the polymer. Figure 4A shows very weak EIS signal at the lowest concentration of PGMA polymer, but the EIS signals increased after increments in PGMA polymer concentrations. At 2.5 mg/mL, the response of the biosensor did not increase much further. For this reason, 1 mg/mL was chosen as the optimal PGMA concentration.

The effect of antibody concentration on immobilization efficiency was examined. Three different concentrations (0.5, 2, and 8 ng/mL) were tested. The lowest concentration had a low signal because of the low amount of anti-KLK4 attachments. As anti-KLK4 concentration increased, the impedimetric signal also increased. At 8 ng/mL, the signal did not increase much further. Thus, 2 ng/mL was used as the optimal amount (Figure 4B).

After optimization of immobilized anti-KLK4 level, the incubation duration of anti-KLK4 was examined. The PGMA polymer-covered electrodes were incubated in anti-KLK4 solution (2 ng/mL) for three different durations (30, 45, and 60 min). Thirty minutes of incubation time was not adequate for anti-KLK4 immobilization. Impedimetric signal increased with incubation time, and it was determined that 45 min was the optimal antiKLK4 duration (Figure 4C). Lastly, KLK4 antigen incubation time was examined. The electrodes immobilized with antiKLK4 antibodies were incubated in KLK4 solution at three different durations (30, 45, and 60 min). As shown in Figure 4D, the impedimetric signals for different incubation durations were similar. As a result, 30 min were used as the optimal immobilization duration for further studies.

### 3.5. Analytical investigations of KLK 4 sensor performance

**Figure 5:**
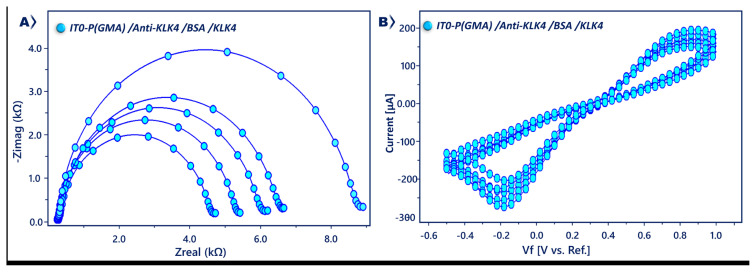
EIS (A) and CVs (B) of modified electrodes in phosphate buffer solutions containing different concentrations of KLK4.

**Figure 6:**
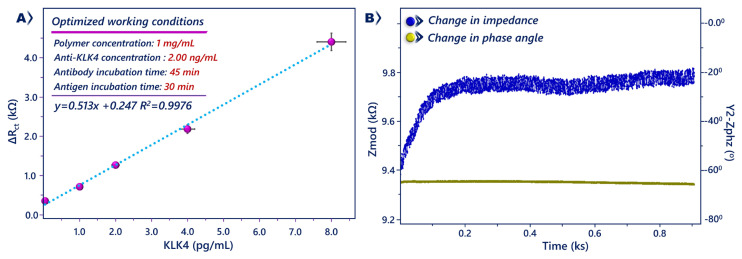
The calibration curve (A) corresponding to impedimetric responses. Error bar = ± standard deviation. Results of impedance recorded at constant frequency (B).

The immunosensor was used to quantify the KLK4 antigen under the optimal experiment conditions. The amount of KLK4 antigen was directly related to the electrochemical signal. The EIS signal increased with increasing KLK4 concentration. The impedimetric response change (ΔR_ct_) was calculated as ΔR_ct_ = R_ct_(KLK4) – R_ct_(BSA), and reflected the level of KLK4. More KLK4 antigen immobilization caused more interference and higher impedimetric responses (Figure 5A). Similarly, the increase in KLK4 concentration caused declines in CV peaks due to more antigen immobilization (Figure 5B). A linear relationship was obtained between ΔR_ct_ and KLK4 concentration, with the calibration curve ranging from 0.04 pg/mL to 8 pg/mL and the linear regression equation of ΔR_ct_ = 0.513 [KLK4] + 0.247 (R^2^ = 0.9976) (Figure 6A). The LOD, determination limit, and sensitivity were computed as 12.21 fg/mL, 40.69 fg/mL, and 2.23 kΩ pg^−1^ mL cm^2^, respectively.

A comparison of the analytical performances of KLK4 detection techniques is presented in Table 1. The immunosensor demonstrates excellent performance by offering a lower detection limit than commercial ELISA kits for the determination of KLK4. Due to its low cost, simplicity, and fast response, this immunosensor is an excellent candidate for the electrochemical analysis of KLK4.

The differences in charge transfer resistance demonstrate the modifications on the electrode surfaces [39,40]. To follow the binding of KLK4 antigen to its anti-KLK4 antibody, a single frequency impedance technique was used. This experiment records the impedance as a function of time at a constant frequency. During this experiment, the binding process between anti-KLK4 and KLK4 is controlled at a repeat time and a total time. The frequency used for analysis is determined from the Bode plot [31,41]. To monitor interaction between KLK4 and anti-KLK4, the device was established at a constant frequency of 22 Hz. This interaction was measured in phosphate buffer (pH 7.4, 50 mM) containing KLK4 antigen. When KLK4 antigen was attached to the immunosensor surface, the impedance started to rise, as seen in Figure 6B. The increase demonstrates time-dependent variations on the electrode surface and binding kinetics between KLK4 and antiKLK4.

**Figure 7:**
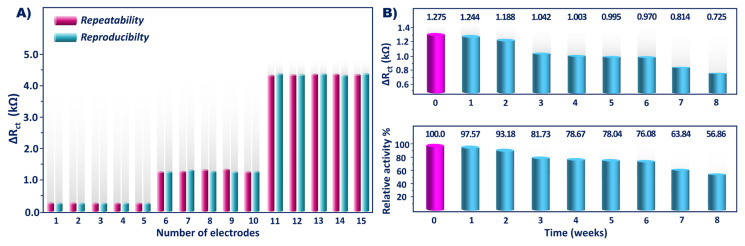
KLK4 biosensor repeatability and reproducibility (A) and stability (B) test results.

Repeatability of the system was tested by successive measurements of KLK4 (0.04, 2, and 8 pg/mL) five times using the constructed bioelectrodes. The relative standard deviation (RSD) was calculated as 5.29 ± 0.03%, 3.40 ± 0.02%, and 0.47 ± 0.01%, respectively. Reproducibility was also studied by measurements of KLK4 (0.04, 2, and 8 pg/mL) five times with 15 different bioelectrodes prepared independently on different days. The RSD was found to be 5.12 ± 0.03%, 2.44 ± 0.01%, and 0.50 ± 0.01%, respectively. The low RSDs of repeatability and reproducibility illustrated the acceptable precision of the biosensor (Figure 7A).

To test for stability, ITO/PGMA/antiKLK4/BSA electrodes were stored at 4 °C for 8 weeks. The signals of the immunoelectrodes were analyzed at the end of each week, and the change in the electrochemical response is shown in Figure 7B. The biosensor retained about 81.73% of its initial response after 3 weeks and decreased gradually to 76.08% and 63.84% after 6 and 7 weeks, respectively. The biosensor was highly stable when stored up to 6 weeks.

**Figure 8:**
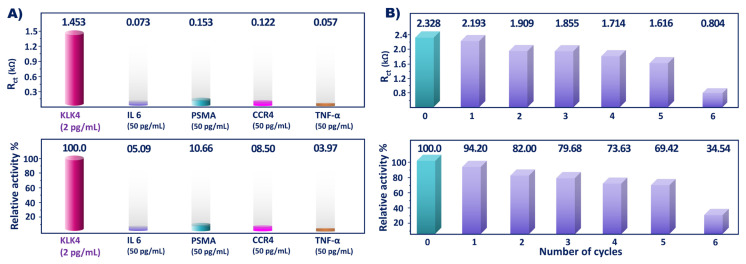
KLK4 biosensor selectivity (A) and reusability (B) test results.

To assess the selectivity of the biosensor, its response to different biomarkers (PSMA, CCR4, TNFα, and IL6) was recorded. In this experiment, the immunosensors were incubated in 50 pg/mL of different biomarkers and KLK4 biomarker (2 pg/mL) solution. The impedimetric responses obtained from this study are shown in Figure 8A. The immunosensor was strongly selective for the KLK4 antigen, and other proteins caused low impedimetric responses. The relative activities of IL6, PSMA, CCR4, and TNF-α biomarkers were found as 5.09 ± 0.03%, 10.66 ± 0.01%, 8.50 ± 0.02%, and 3.97 ± 0.02%, respectively.

Reusability of the bioelectrode was also analyzed by acidic solution (10 mM HCl) treatment. The EIS responses of the regenerated electrode and the remobilized electrode were recorded. After six successive regenerations, the biosensor indicated 69.42% of its initial signal, indicating that the proposed biosensor showed reusability 5 times (Figure 8B).

### 3.6. Spike-in studies

To assess the performance of the suggested sensor in real samples, human sera were used. The matrix effect was examined by adding known amounts (1.0 and 4.0 pg/mL) of the KLK4 antigen to the sample. These samples were analyzed with the biosensor after a 10-fold dilution. The recovery percentages obtained from the spiked samples were in a range from 97.54% to 104.94%. The obtained analysis results are illustrated in Table 2, and the result suggests that the fabricated biosensor could provide an alternative platform for KLK4 screening. Consequently, the KLK4 measurement with the biosensor was reliable, sensitive, and practical.

## 4. Conclusions

In conclusion, a highly sensitive PGMA polymer-based disposable biosensor was designed for the impedimetric analysis of KLK4 biomarker in real sera. PGMA polymer was employed as the matrix material to construct the electrochemical biosensor, and the specific recognition event between the biological proteins was achieved on the PGMA interface. In addition, the PGMA polymer provided a biocompatible environment, and epoxy ends for anti-KLK4 immobilization. Thus, a large surface area was generated on the electrode surface. The electrochemical biosensor exhibited the sensing performance of KLK4 protein with a limit of detection of 12.21 fg/mL and a wide linear measurement range (0.04–8 pg/mL), which demonstrates rapidity, excellent sensitivity, and ease of use. Under optimal conditions, the biosensor was reproducible and sensitive for KLK4 determination in human serum with good accuracy. In addition, the suggested sensor was selective for KLK4 in the presence of other biological markers. In addition, a low-cost analytical platform was created by using an inexpensive ITO substrate as the immobilization platform. Compared to the ELISA kit ($5.87 per test), the immunosensor ($0.05 per test) was significantly less expensive. This low-cost and convenient tool could be an alternative technique for KLK4 analysis, and this strategy might present a viable chance for analysis of real samples.

## Figures and Tables

**Scheme:**
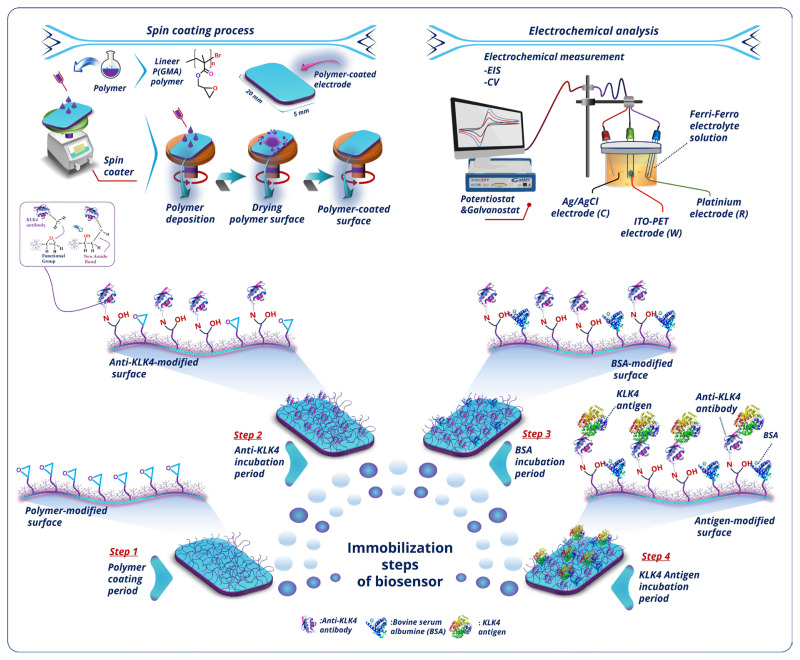
The proposed biosensor fabrication and immobilization mechanism.

**Table 1:** Comparison of the analytical performances of KLK4 detection techniques.

Technique	Linear range (pg/mL)	Detection limit (pg/mL)	Reference
Zinc(II) phthalocyanine tetracarboxylic acid modified biosensor	0.02–15	0.007	[37]
Polythiophene polymer modified biosensor	0.04–30	0.012	[38]
ELISA	35–10^4^	35	RayBiotech
ELISA	312–2×10^4^	120	Abbexa
ELISA	156–10^4^	49	LSBio
ELISA	312–2×10^4^	97	Genochem
ELISA	156–10^4^	39	Bomatik
PGMA-modified biosensor	0.04–8	0.012	Current study

**Table 2:** Real sample analysis results obtained by the fabricated immunosensor.

Human serum	Detected by the biosensor (pg/mL)	Added KLK4 amount (pg/mL)	Total found	% Recovery
**1**	1.32 ± 0.01	1.0	2.29 ± 0.02	98.66 ± 0.01
**1**	1.32 ± 0.01	4.0	5.56 ± 0.02	104.58 ± 0.01
**2**	1.84 ± 0.01	1.0	2.77 ± 0.03	97.54 ± 0.02
**2**	1.84 ± 0.01	4.0	6.11 ± 0.02	104.71 ± 0.03
**3**	2.56 ± 0.01	1.0	3.63 ± 0.03	102.08 ± 0.05
**3**	2.56 ± 0.01	4.0	6.70 ± 0.02	102.11 ± 0.02
**4**	1.88 ± 0.01	1.0	2.85 ± 0.01	99.06 ± 0.01
**4**	1.88 ± 0.01	4.0	6.17 ± 0.02	104.94 ± 0.02
**5**	1.56 ± 0.01	1.0	2.59 ± 0.02	100.99 ± 0.01
**5**	1.56 ± 0.01	4.0	5.55 ± 0.02	99.87 ± 0.01

## References

[1] Afkhami A Hashemi P Bagheri H Salimian J Ahmadi A Impedimetric immunosensor for the label-free and direct detection of botulinum neurotoxin serotype A using Au nanoparticles/graphene-chitosan composite Biosensors and Bioelectronics 2017 93 124 131 10.1016/j.bios.2016.09.059 27665169

[2] Akbari JZ Shayeh JS Yazdian F Yadegari A Hashemi M An electrochemical biosensor for prostate cancer biomarker detection using graphene oxide–gold nanostructures Engineering in Life Sciences 2019 19 3 206 216 10.1002/elsc.201800093 32625003 PMC6999480

[3] Singh S Gill AA Nlooto M Karpoormath R Prostate cancer biomarkers detection using nanoparticles based electrochemical biosensors Biosensors and Bioelectronics 2019 137 213 221 10.1016/j.bios.2019.03.065 31100601

[4] Tezerjani MD Benvidi A Rezaeinasab M Jahanbani S Moshtaghioun SM An impedimeric biosensor based on a composite of graphene nanosheets and polyaniline as a suitable platform for prostate cancer sensing Analytical Methods 2016 8 41 7507 7515 10.1039/C6AY01524G

[5] Crulhas BP Karpik AE Delella FK Castro GR Pedrosa VA Electrochemical aptamer-based biosensor developed to monitor PSA and VEGF released by prostate cancer cells Analytical and Bioanalytical Chemistry 2017 409 29 6771 6780 10.1007/s00216-017-0630-1 29032455

[6] Nimse SB Sonawane MD Song KS Kim T Biomarker detection technologies and future directions Analyst 2016 141 740 755 10.1039/c5an01790d 26583164

[7] Klokk TI Kilander A Xi Z Wæhre H Risberg B Kallikrein 4 is a proliferative factor that is overexpressed in prostate cancer Cancer Research 2007 67 11 5221 5230 10.1158/0008-5472.CAN-06-4728 17545602

[8] Gao J Collard RL Bui L Herington AC Nicol DL Kallikrein 4 is a potential mediator of cellular interactions between cancer cells and osteoblasts in metastatic prostate cancer The Prostate 2007 67 4 348 360 10.1002/pros.20465 17221837

[9] Wilkinson R Woods K D’Rozario R Prue R Vari F Human kallikrein 4 signal peptide induces cytotoxic T cell responses in healthy donors and prostate cancer patients Cancer Immunology, Immunotherapy 2012 61 2 169 179 10.1007/s00262-011-1095-2 21874303 PMC11028920

[10] Nasser NJ Thoms J Soosaipillai A Pintilie M Wang R Human tissue kallikreins: blood levels and response to radiotherapy in intermediate risk prostate cancer Radiotherapy and Oncology 2017 124 3 427 432 10.1016/j.radonc.2017.03.023 28427756

[11] Seiz L Kotzsch M Grebenchtchikov NI Geurts-Moespot AJ Fuessel S Polyclonal antibodies against kallikrein-related peptidase 4 (KLK4): immunohistochemical assessment of KLK4 expression in healthy tissues and prostate cancer Biological Chemistry 2010 391 4 391 401 10.1515/bc.2010.033 20180634

[12] Stephan C Jung K Lein M Diamandis EP PSA and other tissue kallikreins for prostate cancer detection European Journal of Cancer 2007 43 13 1918 1926 10.1016/j.ejca.2007.06.006 17689069

[13] Chen S Cheng YF Voordouw G Three-dimensional graphene nanosheet doped with gold nanoparticles as electrochemical DNA biosensor for bacterial detection Sensors and Actuators B: Chemical 2018 262 860 868 10.1016/j.snb.2018.02.093

[14] Aydın EB Aydın M Sezgintürk MK A highly sensitive immunosensor based on ITO thin films covered by a new semi-conductive conjugated polymer for the determination of TNFα in human saliva and serum samples Biosensors and Bioelectronics 2017 97 169 176 10.1016/j.bios.2017.05.056 28599176

[15] Aydın EB Aydın M Sezgintürk MK Highly sensitive electrochemical immunosensor based on polythiophene polymer with densely populated carboxyl groups as immobilization matrix for detection of interleukin 1β in human serum and saliva Sensors and Actuators B: Chemical 2018 270 18 27 10.1016/j.snb.2018.05.014

[16] Periyakaruppan A Gandhiraman RP Meyyappan M Koehne JE Label-free detection of cardiac troponin-I using carbon nanofiber based nanoelectrode arrays Analytical Chemistry 2013 85 8 3858 3863 10.1021/ac302801z 23384128

[17] Emregul E Kocabay O Derkus B Yumak T Emregul KC A novel carboxymethylcellulose–gelatin–titanium dioxide–superoxide dismutase biosensor; electrochemical properties of carboxymethylcellulose–gelatin–titanium dioxide–superoxide dismutase Bioelectrochemistry 2013 90 8 17 10.1016/j.bioelechem.2012.09.002 23117479

[18] Laschuk NO Easton EB Zenkina OV Reducing the resistance for the use of electrochemical impedance spectroscopy analysis in materials chemistry RSC Advances 2021 11 45 27925 27936 10.1039/D1RA03785D 35480766 PMC9038008

[19] Teles F Fonseca L Applications of polymers for biomolecule immobilization in electrochemical biosensors Materials Science and Engineering: C 2008 28 8 1530 1543 10.1016/j.msec.2008.04.010

[20] Stiriba SE Frey H Haag R Dendritic polymers in biomedical applications: from potential to clinical use in diagnostics and therapy Angewandte Chemie International Edition 2002 41 8 1329 1334 10.1002/1521-3773(20020415)41:8<1329::AID-ANIE1329>3.0.CO;2-P 19750755

[21] Oh WK Kwon OS Jang J Conducting polymer nanomaterials for biomedical applications: cellular interfacing and biosensing Polymer Reviews 2013 53 3 407 442 10.1080/15583724.2013.805771

[22] Galvin CJ Genzer J Applications of surface-grafted macromolecules derived from post-polymerization modification reactions Progress in Polymer Science 2012 37 7 871 906 10.1016/j.progpolymsci.2011.12.001

[23] Azzaroni O Polymer brushes here, there, and everywhere: recent advances in their practical applications and emerging opportunities in multiple research fields Journal of Polymer Science Part A: Polymer Chemistry 2012 50 16 3225 3258 10.1002/pola.26119

[24] Săndulescu R Tertis M Cristea C Bodoki E New materials for the construction of electrochemical biosensors Biosensors-Micro and Nanoscale Applications 2015 1 36 10.5772/60510

[25] Gerard M Chaubey A Malhotra B Application of conducting polymers to biosensors Biosensors and Bioelectronics 2002 17 5 345 359 10.1016/S0956-5663(01)00312-8 11888724

[26] Aydın EB Aydın M Sezgintürk MK Ultrasensitive determination of cadherin-like protein 22 with a label-free electrochemical immunosensor using brush type poly (thiophene-g-glycidylmethacrylate) modified disposable ITO electrode Talanta 2019 200 387 397 10.1016/j.talanta.2019.03.082 31036200

[27] Aydın EB Aydın M Sezgintürk MK Electrochemical immunosensor based on chitosan/conductive carbon black composite modified disposable ITO electrode: an analytical platform for p53 detection Biosensors and Bioelectronics 2018 121 80 89 10.1016/j.bios.2018.09.008 30199712

[28] Oh J Lee JH Koo JC Choi HR Lee Y Graphene oxide porous paper from amine-functionalized poly (glycidyl methacrylate)/graphene oxide core-shell microspheres Journal of Materials Chemistry 2010 20 41 9200 9204 10.1039/C0JM00107D

[29] Aydın M Aydın EB Sezgintürk MK A disposable immunosensor using ITO based electrode modified by a star-shaped polymer for analysis of tumor suppressor protein p53 in human serum Biosensors and Bioelectronics 2018 107 1 9 10.1016/j.bios.2018.02.017 29425857

[30] Clemens G Hands JR Dorling KM Baker MJ Vibrational spectroscopic methods for cytology and cellular research Analyst 2014 139 18 4411 4444 10.1039/C4AN00636D 25028699

[31] Aydın M A sensitive and selective approach for detection of IL 1α cancer biomarker using disposable ITO electrode modified with epoxy-substituted polythiophene polymer Biosensors and Bioelectronics 2019 144 111675 10.1016/j.bios.2019.111675 31518789

[32] Banas A Banas K Furgal-Borzych A Kwiatek WM Pawlicki B The pituitary gland under infrared light–in search of a representative spectrum for homogeneous regions Analyst 2015 140 7 2156 2163 10.1039/C4AN01985G 25574521

[33] Aydın M Aydın EB Sezgintürk MK A highly selective poly (thiophene)-graft-poly(methacrylamide) polymer modified ITO electrode for neuron specific enolase detection in human serum Macromolecular Bioscience 2019 19 8 1900109 10.1002/mabi.201900109 31222894

[34] Tranter G Protein structure analysis by CD, FTIR, and Raman spectroscopies 2017 740 758 10.1016/B978-0-12-409547-2.12099-2

[35] Liu Q Wang H Li H Zhang J Zhuang S Impedance sensing and molecular modeling of an olfactory biosensor based on chemosensory proteins of honeybee Biosensors and Bioelectronics 2013 40 1 174 179 10.1016/j.bios.2012.07.011 22902534

[36] Phelane L Gouveia-Caridade C Barsan MM Baker PG Brett CM Electrochemical determination of tyrosine using a novel tyrosinase Multi-Walled Carbon Nanotube (MWCNT) polysulfone modified glassy carbon electrode (GCE) Analytical Letters 2020 53 2 308 321 10.1080/00032719.2019.1649417

[37] Aydın EB Aydın M Yuzer A Ince M Ocakoğlu Detection of Kallikrein-related Peptidase 4 with a label-free electrochemical impedance biosensor based on a zinc (II) phthalocyanine tetracarboxylic acid-functionalized disposable indium tin oxide electrode ACS Biomaterials Science & Engineering 2021 7 3 1192 1201 10.1021/acsbiomaterials.0c01602 33583176

[38] Aydın EB Aydın M Sezgintürk MK Construction of succinimide group substituted polythiophene polymer functionalized sensing platform for ultrasensitive detection of KLK 4 cancer biomarker Sensors and Actuators B: Chemical 2020 325 128788 10.1016/j.snb.2020.128788

[39] Yang Y Li C Yin L Liu M Wang Z Enhanced charge transfer by gold nanoparticle at DNA modified electrode and its application to label-free DNA detection ACS Applied Materials & Interfaces 2014 6 10 7579 7584 10.1021/am500912m 24734899

[40] Aydın M An ultrasensitive immunosensor based on tri-armed star poly (glycidyl methacrylate) polymer-coated ITO-PET electrode for detection of neuron-specific enolase in human serum International Journal of Environmental Analytical Chemistry 2020 100 4 492 506 10.1080/03067319.2019.1673744

[41] Aydın EB Aydın M Sezgintürk MK Determination of calreticulin using Fe_3_O_4_@ AuNPs core-shell functionalized with PT(COOH)_2_ polymer modified electrode: a new platform for the impedimetric biosensing of cancer biomarkers Sensors and Actuators B: Chemical 2022 367 132099 10.1016/j.snb.2022.132099

